# A Text-Book of Medicine for Students and Practitioners

**Published:** 1887-04

**Authors:** 


**A Text-Book of Medicine for Students and Practitioners.** By ADOLF STRUMPELL, Professor and Director Medical Policlinic at the University of Leipsic. Translated from the third German edition, by Herman F. Vickery, A. B., M. D., and Philip C. Knapp, A M., M. D. With Editorial Notes by Frederick C. Shattuck, A. M., M. D., physician to Massachusetts General Hospital and instructor in theory and practice, Harvard Medical School. With illustrations. New York: D. Appleton & Co., 1, 3 and 5 Bond street.

When a book bearing Appleton's imprint comes to us, we anticipate an enjoyment in the reading of it, and we are seldom disappointed, for their books are nearly always valuable. The present one is an exceptionally good book, and has been adopted as the text-book in the theory and practice of medicine at Harvard. In Germany, Strumpell's work has achieved a great success, having reached a third edition in a short time, and this admirable translation will, we predict, meet with equal favor here. Those chapters which treat of the diseases of the nervous system are models of full, concise and clear instruction. The same characteristics are seen in the other parts of the work, and either to the student or the physician the work is invaluable.

